# Transcriptomic sex differences in sensory neuronal populations of mice

**DOI:** 10.1038/s41598-020-72285-z

**Published:** 2020-09-17

**Authors:** Jennifer Mecklenburg, Yi Zou, Andi Wangzhou, Dawn Garcia, Zhao Lai, Alexei V. Tumanov, Gregory Dussor, Theodore J. Price, Armen N. Akopian

**Affiliations:** 1 Department of Endodontics, University of Texas Health Science Center at San Antonio (UTHSCSA), San Antonio, TX 78229 USA; 2 Greehey Children’s Cancer Research Institute, UTHSCSA, San Antonio, TX USA; 3 Department of Molecular Medicine, University of Texas Health Science Center at San Antonio (UTHSCSA), San Antonio, TX 78229 USA; 4 Department of Neuroscience and Center for Advanced Pain Studies, University of Texas at Dallas School of Behavioral and Brain Sciences, Richardson, TX 75080 USA; 5 Departments of Microbiology, Immunology & Molecular Genetics, University of Texas Health Science Center at San Antonio (UTHSCSA), San Antonio, TX 78229 USA; 6 Department of Pharmacology, The School of Dentistry, University of Texas Health Science Center at San Antonio (UTHSCSA), 7703 Floyd Curl Drive, San Antonio, TX 78229-3900 USA

**Keywords:** Sensory processing, Sexual dimorphism, Pain

## Abstract

Many chronic pain conditions show sex differences in their epidemiology. This could be attributed to sex-dependent differential expression of genes (DEGs) involved in nociceptive pathways, including sensory neurons. This study aimed to identify sex-dependent DEGs in estrous female versus male sensory neurons, which were prepared by using different approaches and ganglion types. RNA-seq on non-purified sensory neuronal preparations, such as whole dorsal root ganglion (DRG) and hindpaw tissues, revealed only a few sex-dependent DEGs. Sensory neuron purification increased numbers of sex-dependent DEGs. These DEG sets were substantially influenced by preparation approaches and ganglion types [DRG vs trigeminal ganglia (TG)]. Percoll-gradient enriched DRG and TG neuronal fractions produced distinct sex-dependent DEG groups. We next isolated a subset of sensory neurons by sorting DRG neurons back-labeled from paw and thigh muscle. These neurons have a unique sex-dependent DEG set, yet there is similarity in biological processes linked to these different groups of sex-dependent DEGs. Female-predominant DEGs in sensory neurons relate to inflammatory, synaptic transmission and extracellular matrix reorganization processes that could exacerbate neuro-inflammation severity, especially in TG. Male-selective DEGs were linked to oxidative phosphorylation and protein/molecule metabolism and production. Our findings catalog preparation-dependent sex differences in neuronal gene expressions in sensory ganglia.

## Introduction

Many inflammatory, neuropathic and idiopathic chronic pain conditions, such as migraine, fibromyalgia, temporomandibular disorder (TMD/TMJ), irritable bowel syndrome (IBS), rheumatoid arthritis, neuropathies, have greater prevalence and/or symptom severity in women as compared to men^1–4^. Additionally, clinical and preclinical data suggest that sex differences in pain prevalence and chronicity is more often encountered for conditions associated with the trigeminal ganglion (TG) system than those that affect the dorsal root ganglia (DRG)^5–8^. Pain symptoms in women with these chronic conditions can change in line with alterations in gonadal hormone concentrations, and some pain conditions decrease in frequency or intensity after menopause^9–12^. These facts and the plethora of clinical and rodent data suggest a critical role for gonadal hormone in regulation of nociceptive plasticity^2,3^.

Gonadal hormones mainly regulate neuronal plasticity via a classic genomic pathway^13^. The genomic pathway involves binding of gonadal hormones to their receptors, nuclear translocation of the gonadal hormone receptor and regulation of transcription either via gonadal hormone responsive elements, such as the estrogen response element^14^ or modulation of other transcription factors^15^. These points suggest that sex differences in gene expression could be detected in nociceptive pathways of mice and humans. For instance, sex differences in expression of genes involved in pain and inflammation, such as *Ntrk1*, *Ccl2*, *Pdgfr*, *Traf3* and *Vegfa* were observed in human tibial nerve^16^. Neuropathic pain conditions are also associated with sex-dependent differential expression of inflammatory genes and transcription factors in human DRG tissues^17^.

Sex differences in gene expression can be identified in RNA sequencing (RNA-seq) experiments. A weakness of RNA-seq from whole tissue is that contributions of different cell types presented in tissue remain unsolved after analysis of RNA-seq data. Thus, when looking for sex differences in gene expression in sensory neurons, previously published conclusions often relied on RNA-seq data generated from whole ganglia. This approach is limited, since whole DRG or TG has many other cell types, and these non-neuronal ganglion cells are numerically predominant compare to sensory neurons. Therefore, the aims of the present study are (1) to examine sex differences in gene expression in enriched sensory neuron populations from both the TG and DRG of mice; and (2) to compare these results with expression in whole ganglia and hind paw of naïve mice. Recent publications indicate that sensory neuronal phenotypes could be influenced by the tissue target of innervation^18^. Accordingly, we also sought to define how innervation target impacts sex differences in gene expression. Overall, our work elucidates DEGs in sensory neurons of the DRG and TG in male and female mice and suggests potential pathways that may play differential roles in nociception and pain chronicity between sexes.

## Results

### RNA-Seq of female and male whole DRG and hind paw tissues

**Figure 1:**
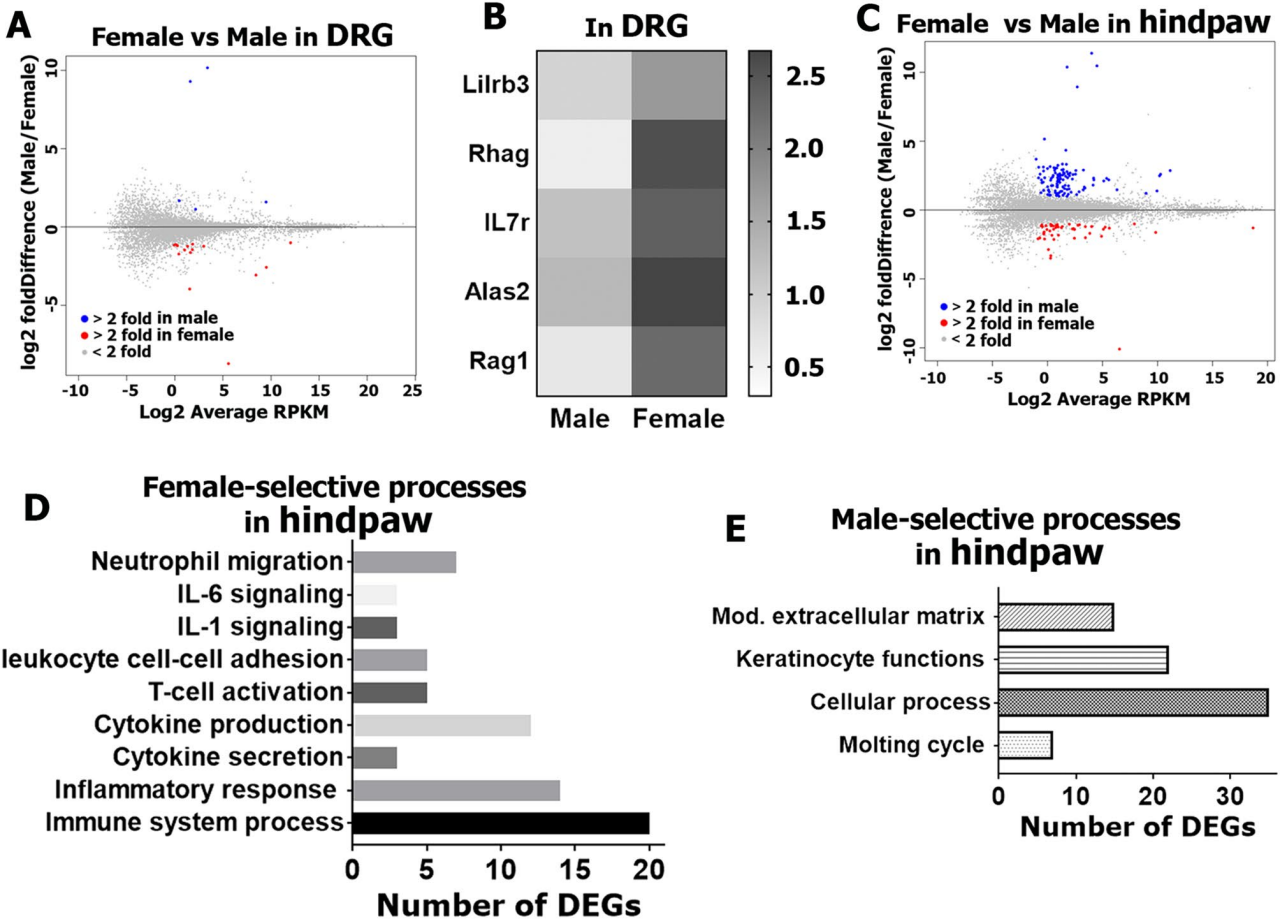
RNA-Seq of female and male whole DRG and hindpaw tissues. Summary plots of DEGs (RPKM > 1; FC > 2; Pval < 0.05) in female and male DRG (**A**) and hindpaw tissues (**C**); (n = 3). Data from mean values of DEGs are shown. Blue dots are male DEGs, red dots are female DEGs. (**B**) Female-predominant DRG DEGs linked to the cell population homeostasis process. Numbers of DEGs involved in female (**D**) and male selective (**E**) biological processes in hindpaws.

Since DRG neurons can interact with cells within the ganglion as well as cells in target tissues like skin to promote pain plasticity^19,20^, we have first evaluated potential sex differences in transcriptional profiles of whole DRG and hind paw tissues in females (all in the estrous phase) versus males (n = 3 per group/sex)^21^. For further analysis of all presented in this manuscript RNA-seq data, we deselected X and Y chromosome-linked genes, such as inactive X specific transcript (*Xist*), ubiquitously transcribed tetratricopeptide repeat gene (*Uty*), lysine (K)-specific demethylase 5D (*Kdm5d*) and eukaryotic translation initiation factor 2, subunit 3, structural gene Y-linked (*Eif2s3y*). Considering this deselection, transcriptomic analysis showed that there were as low as 14 sex-dependent differentially expressing genes (DEGs; RPKM > 1; FC > 2 and Pval < 0.05) in DRG tissues (Fig. 1A, Supplementary excel file). The only DEG in male was *Penk* (proenkephalin), an endogenous opioid, which has anti-nociceptive actions by producing opioid peptides such as enkephalin. Gene clustering according to biological processes using the PANTHER software assigned 5 female-selective DEGs to the cell population homeostasis process (Fig. 1B). This small numbers of female-predominant DRG DEGs were *Lilrb3* (leukocyte immunoglobulin-like receptor subfamily B member 3); *Rhag* (aka CD241; rh-associated glycoprotein); IL7r (aka CD127; interleukin-7 receptor), which is critical for V(D)J recombination; *Alas2* (delta-aminolevulinate synthase 2) genes, which participate in tissue repair^22,23^ and *Rag1* (recombination activating gene 1), which is also vital for T-cell receptor V(D)J recombination. Strengthening selection criteria for sex-dependent DRG DEGs to RPKM > 1; FC > 2 and Padj < 0.05 left a solitary female-predominant DEG—myeloperoxidase (*Mpo*).

**Figure 2:**
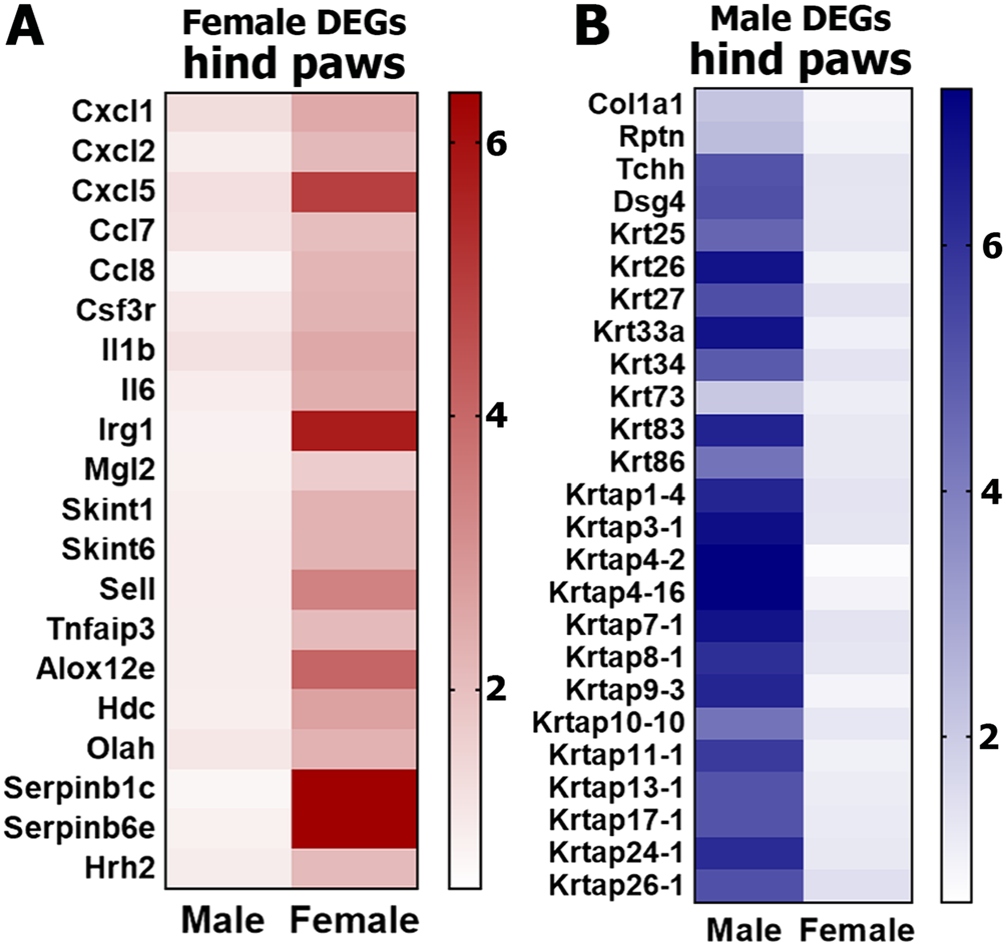
Female and male predominantly expressing hind paw DEGs. Sex-dependent DEGs in hindpaw tissues are: (**A**) inflammatory genes expressing more in female hindpaw; (**B**) epidermal DEGs, especially keratins and keratin-associated protein genes expressing more in male hindpaw.

The number of sex-dependent DEGs (RPKM > 1; FC > 2 and Pval < 0.05) in the hind paw was higher at 142 (Fig. 1C; Supplementary excel file). Female-predominant DEGs in the hind paw covered several biological processes, which were mainly immune cell function related (Fig. 1D). A majority of these female DEGs encode pro inflammatory proteins (Fig. 2A), which are known to sensitize nociceptive pathways. These DEGs include IL-6 and IL-1β cytokines, which are key inflammatory signals in many pain conditions such as fibromyalgia, rheumatoid arthritis, irritable bowel syndrome, etc.^24^; Cxcl1, Cxcl2, Cxcl5, Ccl7 and Ccl8 chemokines, which are known to be involved in nerve injury and chemotherapy-induced neuropathic pain^25–27^. Unlike pro-inflammatory DEGs participating in pain promotion, among female-predominant DEGs was TNF-α regulated *Tnfaip3* (tumor necrosis factor, alpha-induced protein 3), which can inhibit NF-kappa B activation and is critical for limiting inflammation^28^. Female DEGs in hind paws also contained lipid metabolism related genes—*Alox12e* and *Olah*, which increases arachidonic and linoleic acid lipid metabolites that are also known to activate the nociceptive pathway^29,30^. The male-specific DEGs in the hind paw included mostly genes modulating extracellular matrix and keratinocyte functions via increased expression of many members of keratin (*Krt*) and keratin associated protein (*Krtap*) family members (Figs. 1E, 2B). The contribution of these genes to pain pathways has not been studied. Strengthening selection criteria to RPKM > 1; FC > 2 and Padj < 0.05 reduced numbers of sex-dependent hind paw DEGs to 18 female and 33 male-selective DEGs. Importantly, these female DEGs are still linked to immune processes, and male DEGs are associated with keratinocyte functions.

### RNA-Seq of female and male DRG and TG sensory neuronal enriched fractions

Previous reports suggest that sensory neuronal mechanisms, which are manifested at either peripheral or central terminals, could be one of the primary contributors to sexual dimorphisms in chronic pain mechanisms^3^. Our flow cytometry measurements of DRG single-cell suspensions from CGRP^cre-ER/+^/Rosa26^LSL-tDTomato/+^ reporter mice showed that only 2 ± 1.3% (n = 3) of total viable cells were CGRP-cre^+^. Since approximately 40–50% of DRG neurons are peptidergic (i.e. CGRP)^31^, we can estimate that the overall percentage of sensory neurons among all DRG cells is ~ 3–5%. Although, neurons contain more total RNA than non-neuronal DRG cells due to their larger size, RNA-seq data from whole ganglia likely mainly represent the transcriptome of non-neuronal DRG cells, and at the very least under-represents neurons. Hence, to gain better insight into transcriptomic sex differences at the sensory neuron level, we purified DRG and TG sensory neuronal enriched fractions using a Percoll gradient-based approach. Flow cytometry data with a purified sensory neuronal fraction from CGRP^cre-ER/+^/Rosa26^LSL-tDTomato/+^ reporter mouse DRG showed that 35 ± 6.1% (n = 3) of viable cells were CGRP-cre^+^. This indicates that sensory neurons constitute 85–95% of a purified neuronal fraction, since ≈ 40–50% of all DRG sensory neurons are CGRP^+^ as measured by immunohistochemistry^31^.

**Figure 3:**
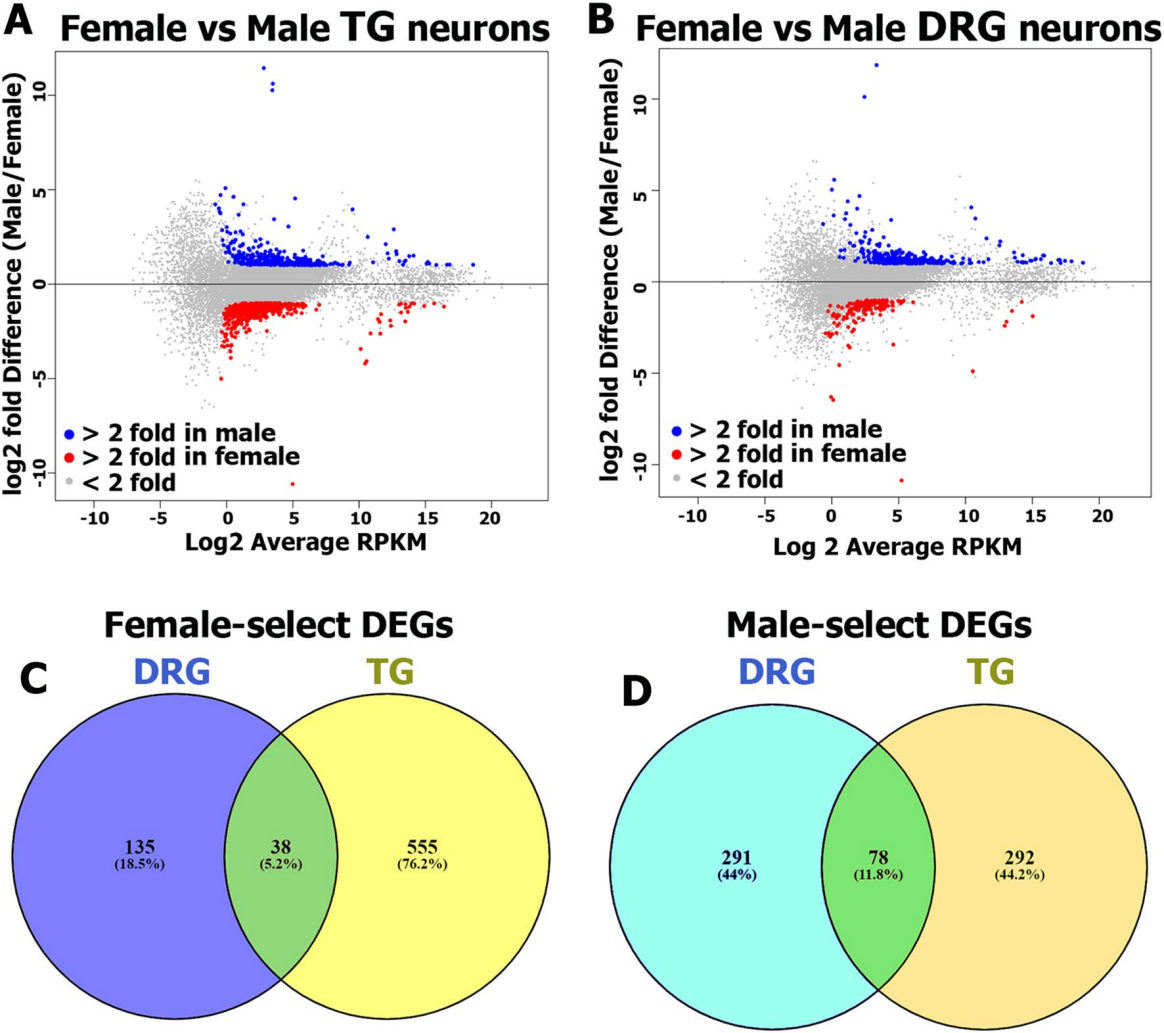
RNA-Seq of female and male DRG and TG sensory neurons. Summary plots of DEGs (RPKM > 1; FC > 2; Pval < 0.05) in female (red dots) and male (blue dots) TG (**A**) and DRG (**B**) neurons; n = 3. Data from mean values of DEGs are shown. (**C**) Venn diagram for RPKM > 1, FC > 2 and Pval < 0.05 female-predominantly expressing TG and DRG sensory neuronal genes. All female DEGs are divided: 555 (76.2%)—only in TG; 135 (18.5%)—only in DRG; and 38 (5.2%)—in both TG and DRG sensory neurons. (**D**) Venn diagram for RPKM > 1, FC > 2 and Pval < 0.05 male-predominantly expressing TG and DRG sensory neuronal genes. All male DEGs are divided: 292 (44.2%)—only in TG; 291 (44%)—only in DRG; and 78 (11.8%)—in both TG and DRG sensory neurons.

Our transcriptomic analysis revealed 963 sex-dependent DEGs (RPKM > 1; FC > 2 and Pval < 0.05) in TG (Fig. 3A, Supplementary excel file), and 540 genes in DRG sensory neurons (Fig. 3B, Supplementary excel file). This DEG numbers were far greater than we observed using a whole DRG approach for RNA-seq (14 DEGs). This suggests that neuronal enrichment increases the ability to discern sensory neuronal-based sex differences.

Female-selective DEGs were greater in number in TG compared to DRG, 594 to 172, respectively. Moreover, only 38 female-selective DEGs were common for DRG and TG (Fig. 3C). In contrast, DRG and TG neurons contained approximately equal numbers of male-selective DEGs, 368 and 369, respectively. Although male-selective DEGs were almost equal in DRG versus TG, only 79 of these DEGs with male-predominant expression were differentially expressed in both TG and DRG neurons (Fig. 3D). Consequently, there were many unique DEGs for both DRG and TG (Fig. 3D). Highly expressed and/or regulated female- and male-selective DEGs in TG and DRG neurons are presented in Table 1 (see also Supplementary excel files). These data suggest that a surprising number of sex differences are not consistent between ganglia, a finding that is consistent with other measures indicating important differences between the TG and DRG^32,33^.

**Table 1:** Highly expressing and/or regulated sex-dependent DEGs in sensory neurons.

Gene ID	Female (RPKM)	Male (RPKM)	FC	Ganglia	Functional cluster
LTbR	9.4134	2.3504	**6.1**	DRG-per	Immune processes
Pdgfb	14.3196	4.5201	**4**	DRG-per	Growth factor and receptors
Ier5	12.1479	4.5225	**2.7**	DRG-per	Transcription
Il33	20.2942	8.0692	**2.5**	DRG-per	Immune processes
Vtn	39.8644	15.8489	**2.5**	DRG-per	Cell adhesion
Cd74	84.1816	38.4553	**2.2**	DRG-per	Immune processes
Tnfrsf11a	13.3066	6.4695	**2.1**	DRG-per	Immune processes
Tnfrsf1a	25.8606	12.3243	**2.1**	DRG-per	Immune processes
Notch3	19.2212	3.5448	**5.5**	TG-per	Transcription activator
Icam1	25.7730	7.6667	**3.5**	TG-per	Extracellular matrix
Cntfr	9.7744	3.3522	**2.9**	TG-per	Growth factor and receptors
Col1a2	55.3372	19.8403	**2.8**	TG-per	Extracellular matrix
Ephb6	34.2972	12.7276	**2.7**	TG-per	Transcription
Socs3	19.2212	7.3801	**2.6**	TG-per	Transcription
Cd74	101.0626	41.2613	**2.5**	TG-per	Immune processes
Nts	1.5979	9.6273	*6.3*	DRG-per	Ligand
Cbr3	5.0846	23.7600	*4.2*	DRG-per	Oxidation–reduction
Rpl13	9.8586	28.0830	*3*	DRG-per	Translation
Med27	8.5363	22.0349	*2.8*	DRG-per	Transcription
Ndufa8	69.0643	164.7326	*2.4*	DRG-per	Cellular respiration
Bud31	26.8360	87.8333	*3.2*	TG-per	Transcription
Cycs	2.8780	6.4099	*3.0*	TG-per	Oxidation–reduction
Glrx3	6.0700	16.7323	*2.7*	TG-per	Oxidation–reduction
Rpl15	24.2493	57.6599	*2.5*	TG-per	Translation
Psmd10	11.2953	25.5359	*2.3*	TG-per	Proteasome complex
Habp4	37.4722	9.8522	**3.8**	DRG-WGA	Transcription
Ajuba	16.1277	4.8867	**3.3**	DRG-WGA	Protein complexes
Cd74	8.0827	2.6312	**3.1**	DRG-WGA	Immune processes
Sox10	61.8918	19.7046	**3.1**	DRG-WGA	Transcription
Fbxo31	82.1134	35.7397	**2.3**	DRG-WGA	Proteasome complex
Fam176b	68.1104	32.7675	**2.1**	DRG-WGA	???
Tmem173	6.9528	27.0744	*4*	DRG-WGA	Immune processes
Alkal2	55.0269	139.9446	*2.5*	DRG-WGA	Ligand for trk receptors
Arxes2	82.6752	172.6092	*2.2*	DRG-WGA	Nervous system develop
Adamts8	6.0444	13.3836	*2.1*	DRG-WGA	Enzyme
Pkib	21.9057	47.3196	*2.1*	DRG-WGA	Channel regulator

*FC* fold change; italic font shows male-selective and bold font represents female-selective DEGs. *DRG-per* are DRG neurons enriched by Percoll; *TG-per* is TG neurons enriched by Percoll; *DRG-WGA* are neurons innervating hindpaw skin and thigh muscle. ???—unknown functional cluster.

### Female-selective DEGs in DRG and TG sensory neuronal enriched fractions

**Figure 4:**
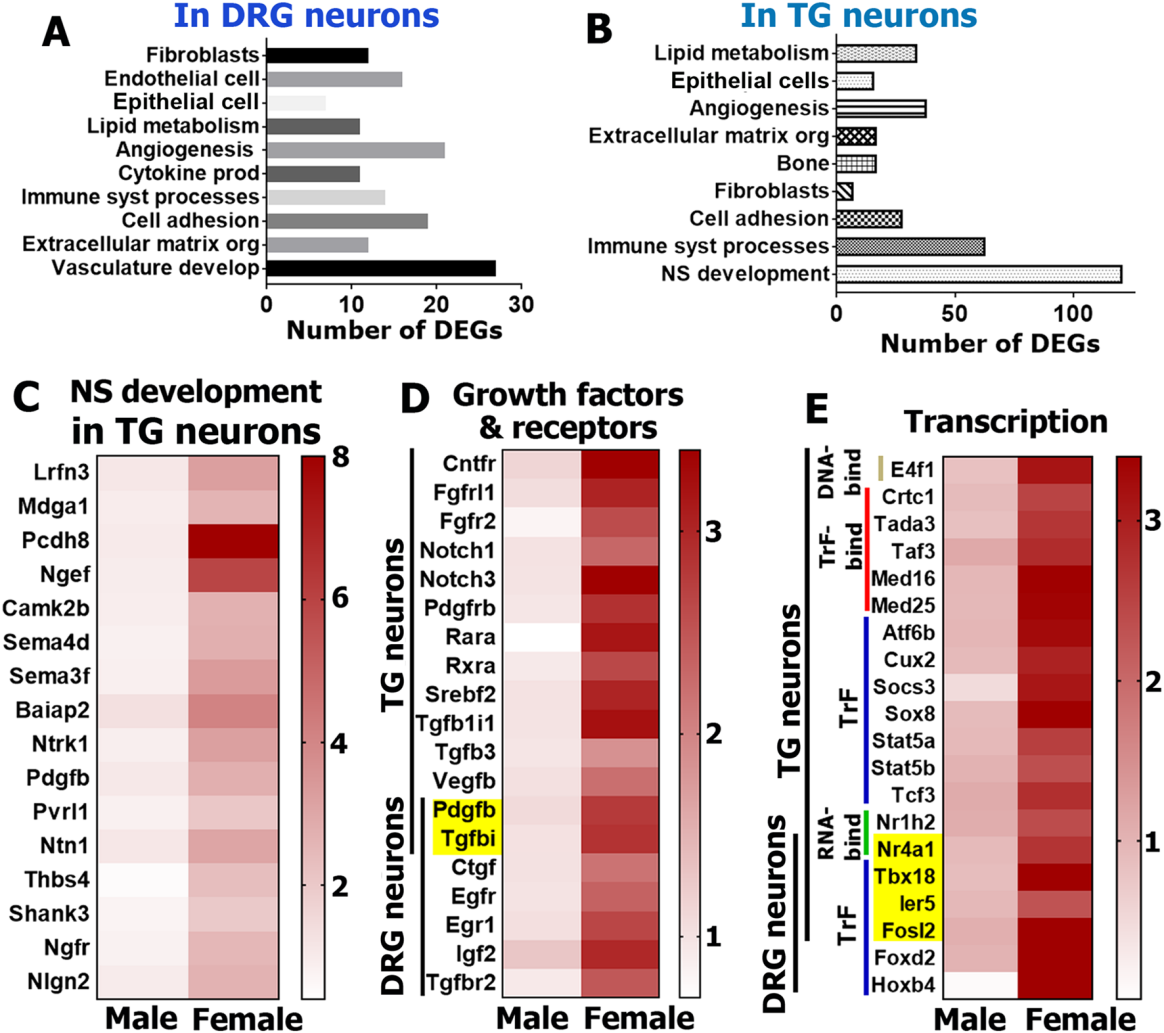
Female-selective DEGs in DRG and TG sensory neurons. Biological processes for female-selective DEGs (RPKM > 1, FC > 2 and Pval < 0.05) in DRG (**A**) and TG (**B**) neurons. (**C**) A set of female-selective DEGs in TG sensory neurons that are involved in the nervous system development process. (**D**) Growth factor and their receptor female-selective DEGs in TG and DRG neurons. (**E**) Transcription machinery female-selective DEGs in TG and DRG sensory neurons. Genes belonging to RNA binding factors are marked as *RNA-bind* (green); DNA binding factors are *DNA-bind* (khaki); transcription factor binding proteins are *TrF-bind* (red); and transcription factors are *TrF* (blue). Yellow boxes on the *panels D* and *E* indicate female-selective DEGs, which were found in both female TG and DRG sensory neurons. Location of TG and DRG neuron female-selective DEGs are marked with vertical lines on the panels (**D**) and (**E**).

We used the PANTHER over-representation test (https://www.pantherdb.org/) to identify distinct biological process clusters for female-selective DEGs from Percoll enriched DRG and TG sensory neuronal fractions (Figs. 4A, 4B). While the TG contains as much as threefold more female-selective DEGs (Fig. 3C), our analysis showed that biological processes linked to female-selective DEGs were similar in DRG vs TG. However, two key differences were noted. First, the TG contains a separate large female-selective DEG cluster involved in nervous system development, including synapse pruning, synaptic vesicle docking, neuron remodeling, neurogenesis, dendritic spine morphogens and regulation of neuronal apoptotic processes (Fig. 4C). Second, similar biological processes, such as extracellular matrix organization, angiogenesis and vascular development, cell adhesion, lipid metabolism, and regulation in epithelial cell, endothelial cell, bone and fibroblasts, include different sets of female-selective DEGs in TG compared to DRG neurons. We also performed a different DEG analysis using PANTHER software, which groups genes according to their functions.

The first cluster contained growth factors and their receptor genes (Fig. 4D). These genes are involved in nervous system development, epithelial cell proliferation, bone development, reposes to lipid, immune system processes, epidermis development, cell–cell adhesion, cell-extracellular matrix adhesion, angiogenesis, etc. (www.uniprot.org). Female DEGs of this cluster were found primarily in TG sensory neurons (Fig. 4D). Among noteworthy growth factor DEGs in TG neurons were *Notch*, *Pdgf* (platelet derived growth factor), *Fgfr* (fibroblast growth factor receptor), *Rara* and *Rxra* (retinoic acid receptors) and *Tgf* (transforming growth factor) families (Fig. 4D). This functional cluster of DEGs are also found in female DRG neurons (Fig. 4D). Thus, *Igf2* (insulin-like growth factor 2) and *Egfr* (epidermal growth factor receptor) are deferentially expressed only in female DRG neurons (Fig. 4D). Additionally, some DEGs belonging to this cluster could be found in both TG and DRG neurons (marked by yellow boxes; Fig. 4D). For instance, platelet derived growth factor, beta polypeptide (*Pdgfb*) and transforming growth factor, beta induced (*Tgfbi*) were enriched in both female TG and DRG neurons.

The second cluster contained female-selective DEGs encoding proteins for the transcriptional and translational machinery (Fig. 4E). These DEGs participate in several biological processes, such as dendritic spine morphogenesis, synapse assembly, morphogenesis of epithelium, regulation of osteoclast proliferation, responses to inflammation, angiogenesis, etc. (www.uniprot.org). Female sensory neurons had DEGs encoding RNA-binding proteins, such as *Nr* family (nuclear receptors); DNA-binding proteins, such as *E4f1* (E4F transcription factor 1); and transcription factor-binding co-factors, such as the *Med* family, *Crtc1* (CREB regulated transcription coactivator 1) and *Tada3* (transcriptional adaptor 3). Some of these transcription factors, notably *Stat5* (signal transducer and activator of transcription 5), *Socs3* (suppressor of cytokine signaling 3), *Sox 8* (sex determining region Y-box 8) and *Hoxb4* (homeobox beta4), have been implicated in regulation of pain chronicity (Fig. 4E)^34,35^. It is also possible that higher expression of these DEGs in female sensory neurons could play a protective role during pain conditions^35–37^.

**Figure 5:**
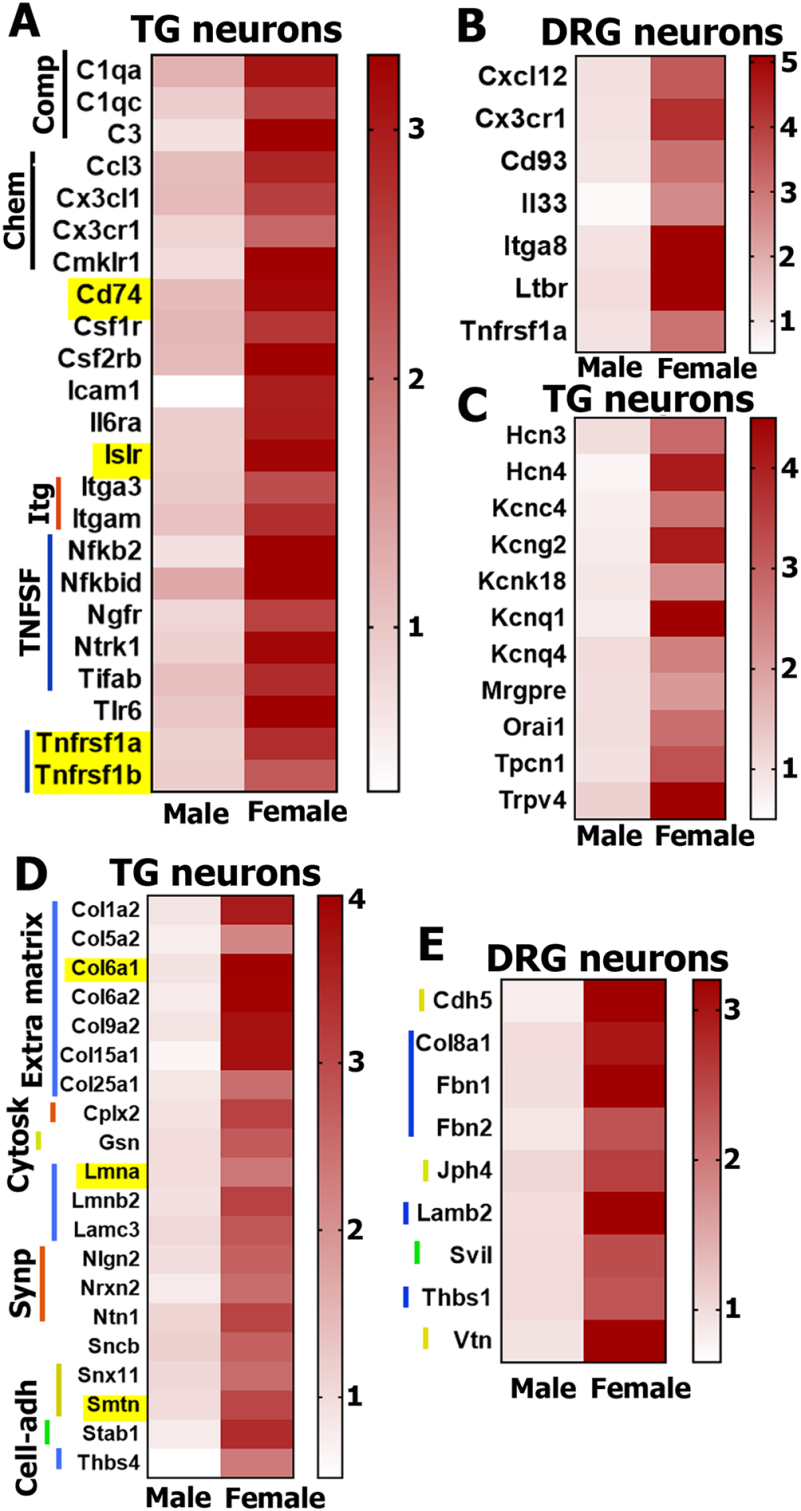
Female-selective DEG functional clusters in DRG and TG sensory neurons. Inflammatory DEGs in female TG (**A**) and DRG (**B**) sensory neurons. Genes belonging to the complement family are marked as *Comp*; chemokines and their receptors are *Chem*; the integrins are *Itg*; and the tumor necrosis factor super family genes are *TNFSF*. Yellow boxes on the panel *A* indicate DEGs in both female TG and DRG sensory neurons. (**C**) Channel genes predominantly expressing in female TG sensory neurons. Structural protein genes expressing higher in female TG (**B**) than in DRG (**C**) sensory neurons. Structural protein DEGs belonging to extracellular matrix are marked as *Extra matrix*; synaptic machinery is *Synp*; cell adhesion is *Cell-adh*; and cytoskeleton are *Cytosk*. Yellow boxes on the panel (**D**) indicate DEGs identified in both male TG and DRG sensory neurons.

The third cluster of female-selective DEGs were genes involved in immune system processes (www.uniprot.org; Fig. 5A,B). Like growth factor genes, these DEGs were more prominent in TG than DRG neurons. DEGs found enriched in female TG included members of the complement system (*C1q* and *C3*), which is part of the innate immune system and is involved in nociception regulation^38^. Both TG and DRG neurons contained female-selective DEGs belonging to the chemokine system (*Cx3cl1*, *Cx3cr1*, *Ccl3*, *Cxcl12* and *Cmklr1*), which regulate monocyte, macrophage and natural killer (NK) cell migration/trafficking^39^ and neuropathic pain^40–42^; the cytokine system (*IL6ra* and *IL33*); the integrin system (*Itga8*, *Itga3* and *Itgam*); the tumor necrosis factor super family members (*Tnfrsf1a, Tnfrsf1b, Ltbr, Ngfr* and *Ntrk1*) and their effectors (*Nfkb2, Nfkbid, Tifab and Icam1*), many of which are key regulators of pain chronicity^24,38,42–44^; and cluster differential factor (*CD74* and *CD93*) (Fig. 5A,B). Some of these DEGs, marked with yellow boxes, were found in both TG and DRG neurons (Fig. 5A).

The fourth functional cluster of female-selective sensory neuronal DEGs consists of channel, metabotropic receptor and transmembrane protein genes (Fig. 5C). They were exclusively enriched in female TG, but not female DRG neurons, and play key roles in nervous system function/development. Almost all these genes, including *Hcn* (hyperpolarization-activated, cyclic nucleotide-gated K^+^) and *Kcn* families (potassium voltage-gated channels), as well as *Orai1* (ORAI calcium release-activated calcium modulator 1) and *Trpv4* (transient receptor potential cation channel, subfamily V, member 4), are known to modulate nociceptor excitability, transmission of nociceptive information and regulation of pain chronicity^45–47^.

The fifth and final cluster of female-predominantly expressing genes in TG and to a lesser extent in DRG sensory neurons were structural proteins (Fig. 5D,E). These DEGs represent extracellular matrix proteins, such as *Col* (collagen), *Lmn* (lamin), *Lam* (laminin), *Thbs* (thrombospondin) and *Fbn* (fibrillin) families; synaptic machinery proteins, such as *Cplx2* (complexin 2), *Nrxn2* (neurexin II) and *Nlgn2* (neuroligin 2); and a few cell adhesion and cytoskeletal proteins, including *Smtn* (smoothelin), *Stab1* (stabilin 1), *Svil* (supervillin), *Vtn* (vitronectin) and *Cdh5* (cadherin 5).

### Male-selective DEGs in DRG and TG sensory neuronal enriched fractions

**Figure 6:**
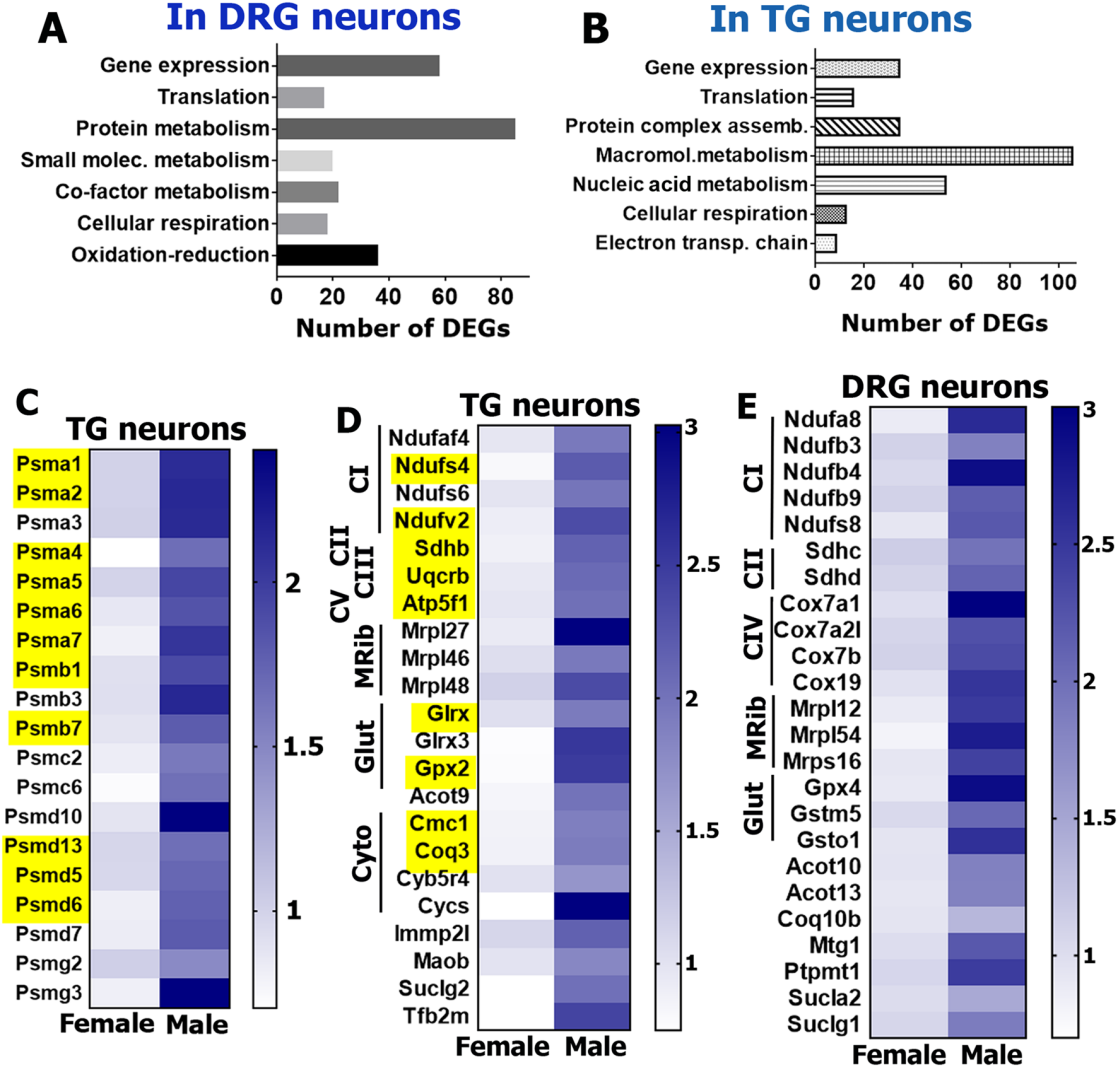
Male-selective DEGs in DRG and TG sensory neurons. Biological processes for male-selective DEGs (RPKM > 1, FC > 2 and Pval < 0.05) in DRG (**A**) and TG (**B**) neurons. (**C**) Proteasome subunit encoding DEGs are male-enriched in TG sensory neurons. (**D**) Male-predominant mitochondrial genes in TG sensory neurons. (**E**) Male-predominant mitochondrial genes in DRG sensory neurons. Yellow boxes on the panels *C* and *D* indicate DEGs in both male TG and DRG sensory neurons. Genes belonging to oxidative phosphorylation complexes I–V are marked as *CI–CV*; mitochondrial ribosomal proteins are *MRib*; the anti-oxidative glutathione pathway are *Glut*; and the cytochrome pathway are *Cyto*.

Although there were many unique male-selective DEGs for TG or DRG neurons (Fig. 3D), the PANTHER over-representation test (https://www.pantherdb.org/) grouped these DEGs into similar biological process clusters for TG and DRG neurons (Fig. 6A, B). These biological processes, including oxidation–reduction, cellular respiration, macromolecule and protein metabolism, translation and gene expression, were represented by a set of distinct male-selective DEGs in TG versus DRG neurons. Similar to how we organized female-selective DEGs, male-selective DEGs were clustered by enriched functional families using the PANTHER classification system (https://www.pantherdb.org/). This analysis highlighted four main DEG clusters in male TG and DRG neurons.

The first cluster consisted of DEGs encoding proteasome subunits and assembly chaperones (Fig. 6C). These DEGs were found in both TG and DRG neurons (yellow boxes; Fig. 6C); and are closely involved in a vast array of biological functions related to protein metabolism and complex assembly (www.uniprot.org; Fig. 6A,B). Proteasomes could play key roles in pain processing, since they contribute to recovery from tissue injury^48^, from nerve damage^49^ and resolution of inflammation^50^.

The second cluster consisted of mitochondrial genes, which had higher levels of expression both in the male TG (Fig. 6D) and DRG sensory neurons (Fig. 6E). These mitochondrial genes can be divided into several sub-groups according to their involvement in different biological processes, such as oxidation–reduction, electron transport chain, cellular respiration, and co-factor and small molecule metabolism (www.uniprot.org; Fig. 6D,E). One of the DEG sub-groups in male TG and DRG sensory neurons represents the oxidative phosphorylation proteins, which are also critical for cellular respiration and recovery from tissue and nerve injury^51,52^. Genes belonging to all five main complexes of the oxidative phosphorylation pathway—reduced nicotinamide adenine dinucleotide (NADH) dehydrogenase (complex I), succinate dehydrogenase (complex II), cytochrome c reductase (complex III), cytochrome c oxidase (complex IV) and adenosine triphosphate (ATP) synthase (complex V)—had male-predominant expression in both TG and DRG sensory neurons (Fig. 6D,E). Another male sensory neuronal DEG sub-group contained coenzymes and cofactors, such as *Cycs* (cytochrome c, somatic), *Cyb5r4* (cytochrome b5 reductase 4), *Cmc1* (cyclooxygenase (COX) assembly mitochondrial protein 1), Coq3 (coenzyme Q3 methyltransferase), *Coq10a* (coenzyme Q10A), *Moab* (monoamine oxidase B), *Sucl* family (succinate-CoA ligase) and the *Acot* family (acyl-coenzyme A (CoA) thioesterase), supporting the oxidative phosphorylation pathway. Genes involved in protection from oxidative damage, *Gpx* (glutathione peroxidase), *Glrx* (glutaredoxin) and the *Gst* families (glutathione S-transferase) were also higher in male sensory neurons (Fig. 6D,E). Finally, mitochondrial protein anabolism genes also showed higher expression in males including *Mterfd* (Mitochondrial Transcription Termination Factors), *Rpp30* (ribonuclease P/MRP 30 subunit), *Tfb2m* (transcription factor B2, mitochondrial) *Mtg1* (mitochondrial ribosome-associated GTPase 1), and the *Mrpl* and *Mrps* families.

**Figure 7:**
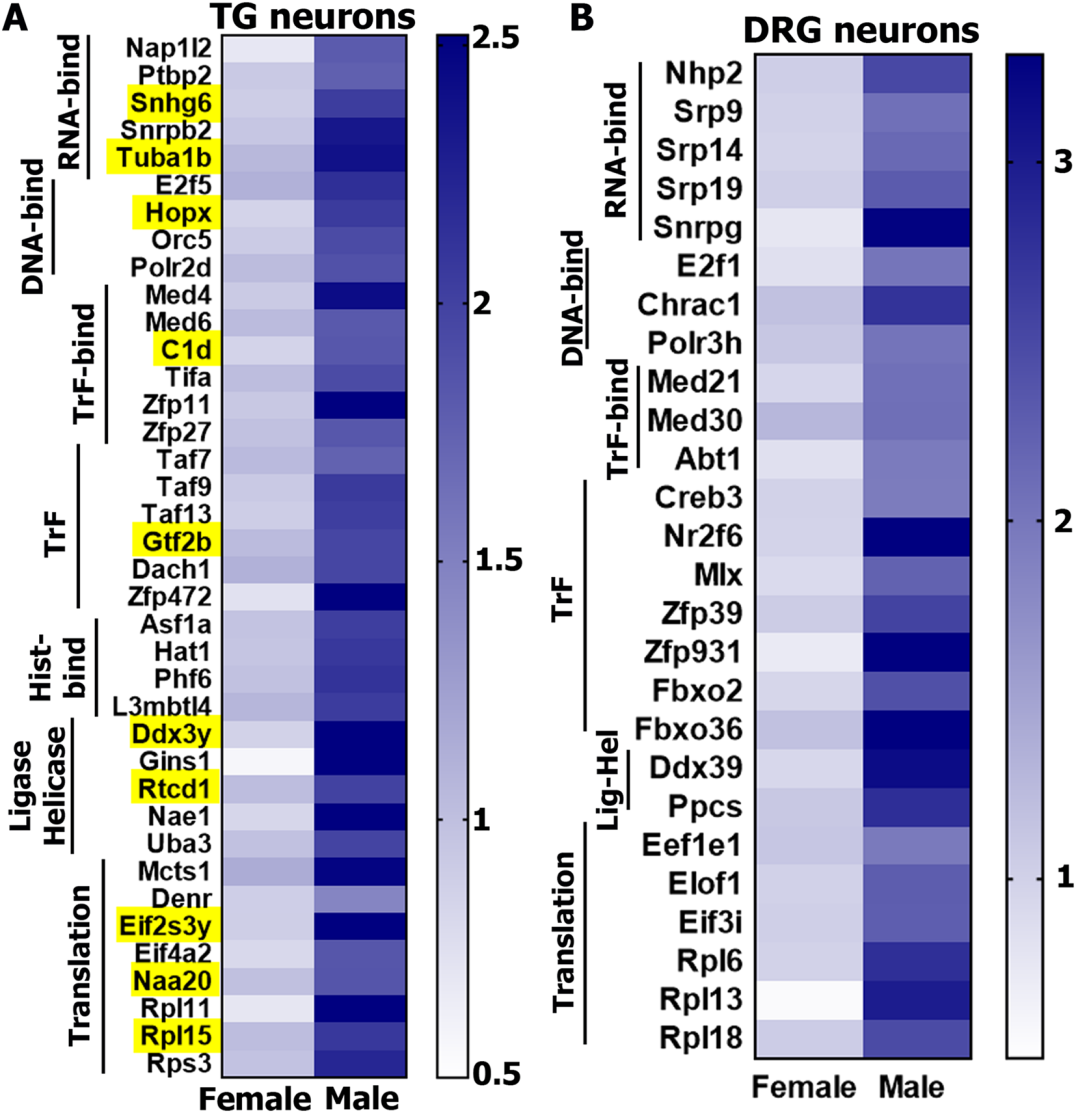
Male-selective DEG functional clusters in DRG and TG sensory neurons. Male-predominant transcription and translation machinery genes in TG (**A**) and DRG (**B**) sensory neurons. Yellow boxes on the panel *A* indicate DEGs in both male TG and DRG sensory neurons. Genes belonging to RNA binding factors are marked as *RNA-bind*; DNA binding factors are *DNA-bind*; transcription factor binding proteins are *TrF-bind*; transcription factors are *TrF*; histone binding proteins are *His-bind*; ligases and helicases are *Ligase Helicase* or *Lig-Hel*; and translation machinery proteins are *Translation*.

The third and fourth clusters of DEGs in male sensory neurons related to non-mitochondrial transcription and translation machinery (Fig. 7A,B). Interestingly, these male-selective DEGs did not overlap with female-selective DEGs representing transcription machinery (Fig. 4E). Transcription machinery genes with higher expression in males could be separated into several sub-groups: genes of RNA-binding proteins, such as *Snhg6* (small nucleolar RNA host gene 6) and *Snrpb2* (U2 small nuclear ribonucleoprotein B); DNA-binding proteins, such as *Polr2d* (polymerase (RNA) II (DNA directed) polypeptide D), *Tcea2* (transcription elongation factor A (SII), 2) and *Orc* (origin recognition complex); transcription factor-binding co-factors, such as *C1d* (C1D nuclear receptor co-repressor), *Tifa* (TRAF-interacting protein with forkhead-associated domain) and *Med* (mediator complex subunit); histone-binding proteins, such as *Hat1* (histone aminotransferase 1), *Asf1a* (anti-silencing function 1A histone chaperone) and *L3mbtl4* (L3MBTL4 histone methyl-lysine binding protein); ligases and helicases, such as *Rtcd1* (RNA 3′-Terminal Phosphate Cyclase) and *Nae1* (NEDD8 activating enzyme E1 subunit 1); and, finally, general transcription factors, such as the *Zfp* family (zinc finger proteins), *Gtf2b* (general transcription factor IIB) and the *Taf* family (TATA-box binding protein associated factors). In contrast to female DEGs in this class of genes (Fig. 4E), male DEGs include histone-binding proteins and ligases/helicases (Fig. 7A,B). Male-enriched mRNAs in the translation machinery family were mainly composed of genes encoding *Eif* family (eukaryotic translation initiation factors), *Naa20* (N(alpha)-acetyltransferase 20, NatB catalytic subunit), and *Rpl* and *Rps* families (ribosomal proteins).

### RNA-Seq of female and male sensory neurons innervating hindpaw skin and thigh muscle

Recent reports indicate that peripheral tissue innervation influences sensory neuron phenotype and potentially sensory neuron plasticity^18^. We sought to clarify whether the transcriptome of sensory neurons innervating hindpaw skin or thigh muscle in males or females would be distinct from data generated on Percoll enriched fraction of DRG neurons. To do this, we injected WGA-488 simultaneously into left and right hindpaws as well as left and right thighs of mice. The rational for combine injection into skin and muscle was to increase a number of WGA-488^+^ neurons. These injections were performed for females and males (n = 4 each group). Two days post-injection L3-L5 DRG were dissected and generated single-cell suspensions were generated. A mixture of skin and thigh innervating neurons were isolated by double sorting WGA-488^+^ neurons using a 130 μm nozzle. The use of the 130 μm nozzle on a FACS sorter is essential to avoid losing medium-to-large (> 35 μm) neurons during the sorting procedure. Each group (female or male) had 4 samples and 6 DRG (L3-L5 levels from left and right sides) within sample; and this sorting procedure generated 1,500–2,500 WGA-488^+^ neurons for each sample. cDNA library from extracted RNA was synthesized using PCR-based SmartSeq II protocol.

**Figure 8:**
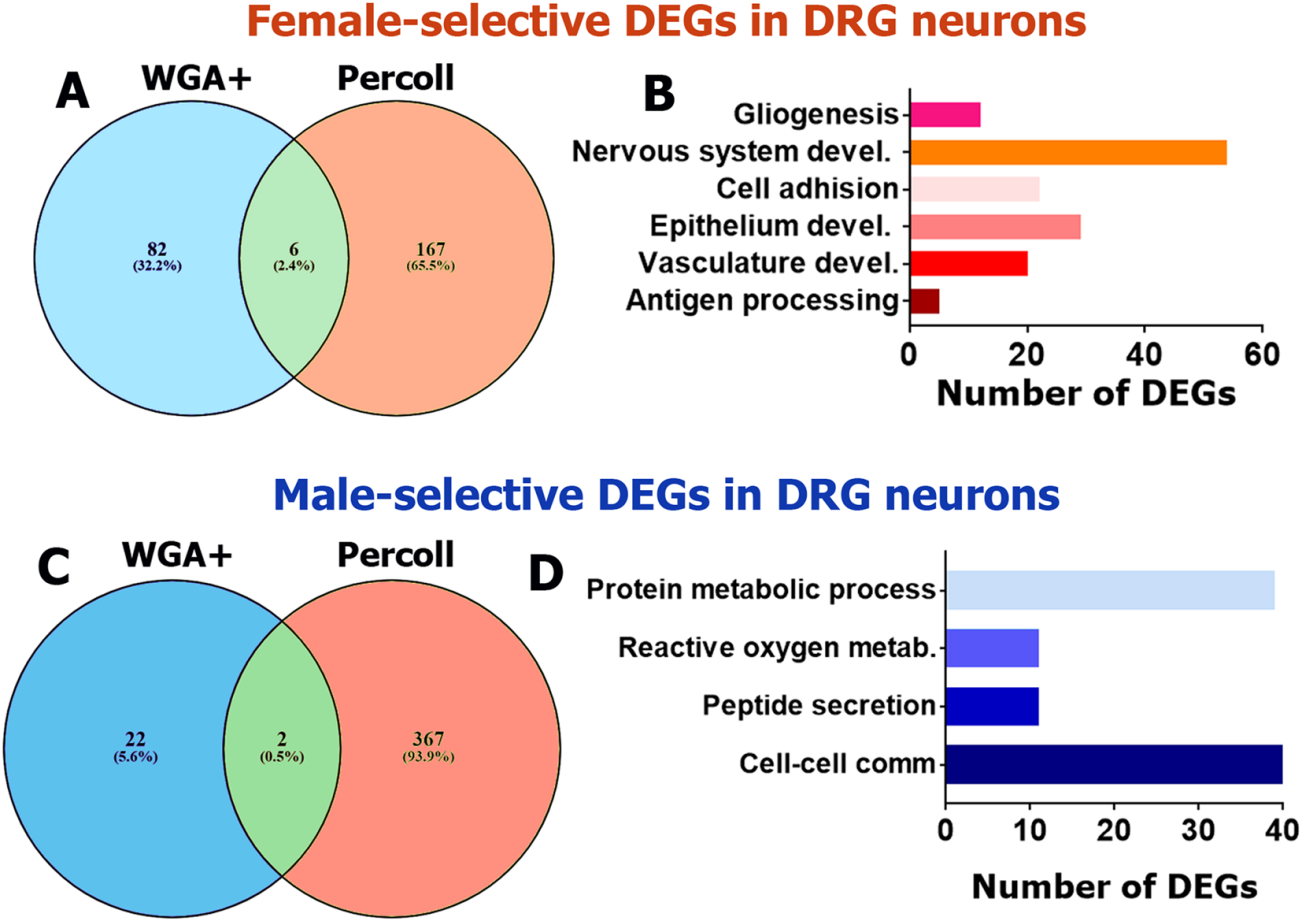
Sex-dependent DEGs in DRG neurons innervating hindpaw skin and thing muscle. Venn diagram for RPKM > 1, FC > 2 and Pval < 0.05 female (**A**) and male-predominantly (**C**) expressing WGA-488-labeled DRG neurons innervating hindpaw skin and thigh (WGA^+^) and Percoll purified DRG neurons (Percoll). All female DEGs are divided: 82 (32.2%)—only in WGA^+^; 167 (65.5%)—only in Percoll purified; and 6 (2.4%)—in both sensory neuron preparations. All male DEGs are divided: 22 (5.6%)—only in WGA^+^; 367 (93.9%)—only in Percoll purified; and 2 (0.5%)—in both sensory neuron preparations. Biological processes for female (**B**) and male-selective (**D**) DEGs (RPKM > 1, FC > 2 and Pval < 0.05) in DRG neurons innervating hindpaw skin and thing muscles.

Our transcriptomic analysis showed that there are 88 female-selective DEGs (RPKM > 1; FC > 2 and Pval < 0.05) in WGA^+^ neurons. Six DEGs, *Jub* (LIM domain-containing protein ajuba), *Cd74* (CD74 antigen), *Abi3bp* (ABI gene family, member 3 (NESH) binding protein), *Bgn* (biglycan), *Mxra8* (matrix-remodeling associated 8) and *Islr* (immunoglobulin superfamily containing leucine-rich repeat), are identified in WGA^+^ and Percoll purified DRG neurons (Fig. 8A). Moreover, *Cd74* and *Islr* were female-selective DEGs in Percoll purified TG neurons as well (Table 1). *Cd74* and *Jub* play a critical role in pro-inflammatory processes (www.uniprot.org). *Abi3bp*, *Islr*, *Bgn* and *Mxra8* contribute to cell–cell adhesion and extracellular matrix organization processes (www.uniprot.org). The PANTHER over-representation test (https://www.pantherdb.org/) groups these 88 female-selective DEGs into antigen processing and nervous system development biological processes. Analysis of 285 female-predominant DEGs selected according to RPKM > 1; FC > 1.5 and Pval < 0.05 revealed additional biological processes, such as gliogenesis, cell adhesion, epithelium and vasculature development (Fig. 8B).

RNA-seq analysis indicates that only 24 DEGs (RPKM > 1; FC > 2 and Pval < 0.05) in WGA^+^ neurons could be classified as male-selective. Among these 24 male-predominant DEGs, *Pkib* (protein kinase inhibitor beta, cAMP dependent, testis specific) and *Arxes2* (adipocyte-related X-chromosome expressed sequence 2) were common for both WGA^+^ and Percoll purified DRG neurons (Fig. 8C). *Pkib* is a potent competitive inhibitor of cAMP-dependent protein kinase activity (www.uniprot.org), and *Pkib* up-regulation could potentially lead to anti-pain effect in inflammatory pain conditions. However, although *Pkib* is expressed in DRG neurons at high levels^53^, there is no literature its involvement in pain. *Arxes2* is also highly expressed in DRG neurons^53^, but its function in neurons is unknown. The overlap between male-selective DEGs in WGA^+^ DRG neurons and Percoll purified TG neurons was wider than between DEGs in WGA^+^ DRG neurons and Percoll purified DRG neurons; and includes *Ptbp2* (polypyrimidine tract binding protein 2), *Dcun1d1* (DCN1, defective in cullin neddylation 1, domain containing 1), *Pcdhb20* (protocadherin beta 20), *Pkib*, *Tifa* (TRAF-interacting protein with forkhead-associated domain), *Stk32a* (serine/threonine kinase 32A), *Lipo1* (lipase, member O1) and *Serpina3i* (serine (or cysteine) peptidase inhibitor, clade A, member 3I). A low number of male-selective DEGs (24) was not enough for linking them to statistically significant biological process using the PANTHER over-representation test (https://www.pantherdb.org/). To overcome this, we decreased strength of selection criteria to RPKM > 1; FC > 1.5 and Pval < 0.05. This revealed 122 male-selective DEGs that can be linked to biological processes (Fig. 8D). Two biological processes – reactive oxygen and protein metabolism—were common for RNA-seq analysis of DEGs identified in Percoll purified neurons and WGA^+^ neurons (Figs. 6A,B, 8D).

**Figure 9:**
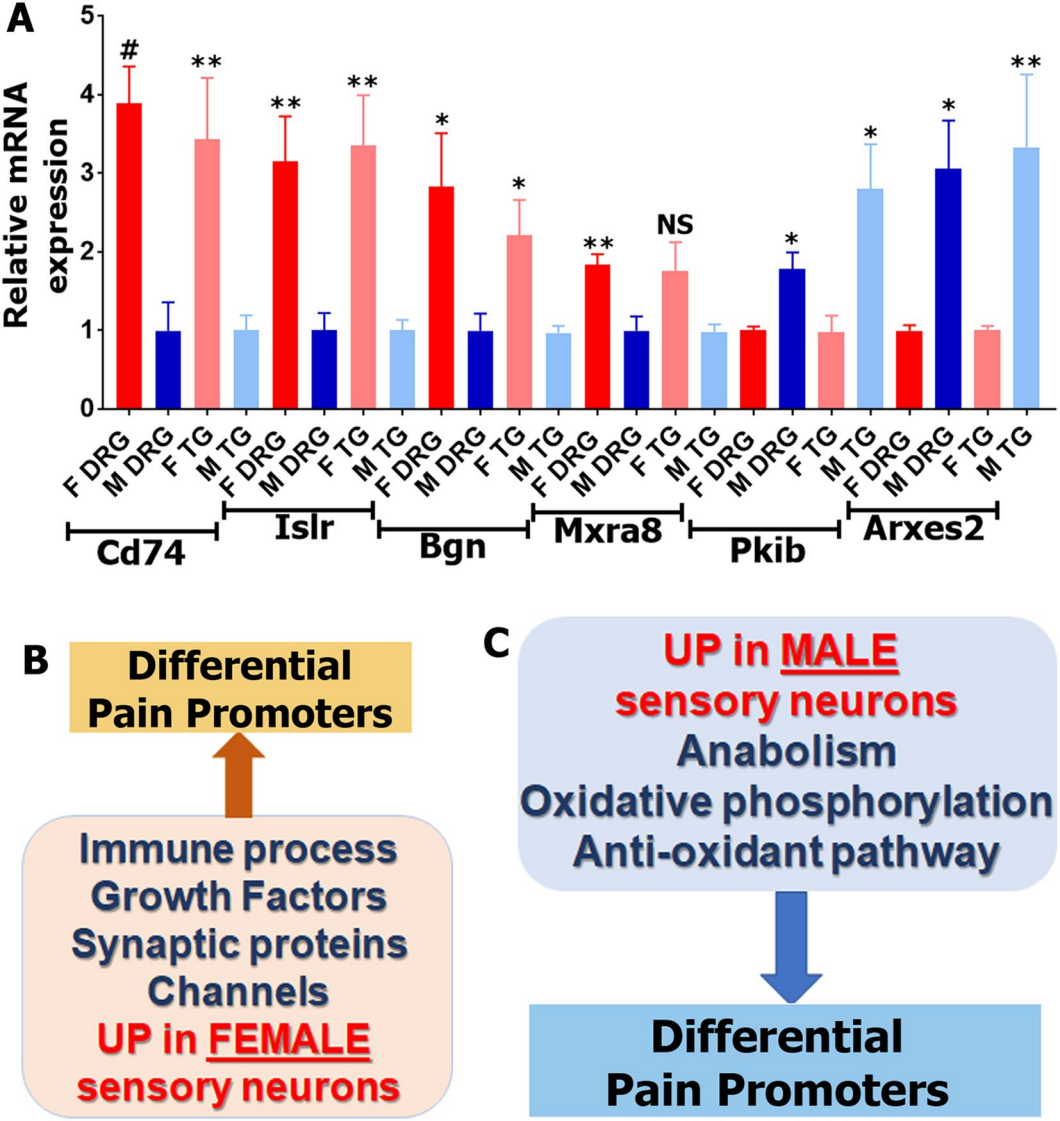
Quantitative RT-PCR validation of RNA-seq identified sex-dependent DEGs in sensory neurons. (**A**) Quantitative RT-PCR for 6 sex-dependent sensory neuronal DEGs. Female (F DRG) and male (M DRG) DRG sensory neurons are identified by back-tracing from hindpaw and thigh. Female (F TG) and male (M TG) TG sensory neurons are purified using Percoll gradient. Statistic is un-paired *t*-test (NS p > 0.05; *p < 0.05; **p < 0.01; # p < 0.0001; n = 4). Name of DEGs are indicated. Schematic of female (**B**) and male (**C**) sensory neuronal DEGs clusters that could explain difference in pain promoters in female versus male mice.

Finally, we used quantitative RT-PCR for validation of a set of DEGs that have been identified in neurons prepared by all procedures: Percoll from TG, Percoll from DRG as well as WGA-labeled DRG neurons. Accordingly, we have selected *Cd74*, *Islr*, *Bgn*, *Mxra8*, *Pkib* and *Arxes2*. RNA was prepared from female and male TG neurons enriched by Percoll. Another RNA preparation was from DRG neurons labeled by back-tracing WGA-488 from both hindpaws and thigh muscles. Figure 9A shows that *Cd74, Islr, Bgn* and *Mxra8* have female-predominant expression, while *Pkib* and *Arxes2* are higher expression levels in male sensory neurons in both Percoll enriched TG neurons and WGA-488^+^ DRG neurons.

## Discussion

There is now a strong body of clinical and basic science studies demonstrating sex differences in the mechanisms contributing to the development and/or maintenance of pain in males and females. There is epidemiological evidence that males and females may be prone to different types of chronic pain disorders^1–4^. Sex differences in gene expression in nociceptive pathways, including sensory neurons, of humans and animals could potentially elucidate mechanistic reasons for why these differences exist. Accordingly, we aimed to identify sex differences in gene expression in sensory neurons. These differences could also be influenced by several factors, aside from biological sex. First, DRG and TG have very different physiological functions and gene expression profiles^33^. Second, the preparation method of sensory neurons can impact the RNA-seq outcome. Third, the innervated tissue can contribute to sensory neuronal phenotype, or different tissues can be innervated with distinct subsets of sensory neurons^18^. We confirm that only a small proportion of cells of DRG are sensory neurons, and the remaining cells are non-neuronal. Hence, RNA-seq data analysis of whole DRG (or TG) will reflect a strong influence of non-neuronal cells on the transcriptome. Analysis of our data showed that hindpaw and especially whole DRG of naïve mice have relatively few sex differences at the transcriptomic level (Figs. 1, 2). Consequently, we examined transcriptomic differences on isolated and purified TG and DRG sensory neuronal fractions from naïve females (estrous phase) and males where we found prominent sex differences that may have important implications for understanding how pain mechanisms differ in men and women (Figs. 3–7). Furthermore, we isolated female and male mouse sensory neurons innervating hindpaw skin and thigh muscle; and used RNA from those cells for RNA-seq and analysis (Figs. 8, 9). These experiments showed that certain sets of sex-dependent DEGs are influenced by tissue innervation. There could be two explanations for this phenomenon: first, tissue innervation could influence sensory neuron transcriptomics^18^; or second, the neuronal subsets innervating different tissues could be distinct to begin with and therefore, are guided to different targets. The latter notion is supported by single cell sequencing of back labelled neurons from the colon^54^.

Groups of female-predominant DEGs generated from Percoll purified DRG and TG neurons, and skin or thigh innervating L3–L5 DRG neurons showed low overlap. The highest numbers (593) of such DEGs (RPKM > 1; FC > 2; Pval < 0.05) were identified in Percoll purified TG neurons, and the lowest (88) were in DRG neurons innervating skin and thigh (Figs. 3C,8A). Nevertheless, there is substantial overlap in biological processes linked to these DEGs (Figs. 4A,B, 8B). We observed broadly higher expression of DEGs involved in immune processes in Percoll purified female DRG and TG neurons as well as DRG neurons innervating the skin and thigh muscle. These female-selective DEGs showed preferential expression in female DRG and TG, with notably larger differences in the TG neurons. In this cluster, the complement system^38^; certain chemokines regulating monocyte, macrophage and NK cell migration/trafficking^40–42^; IL-1, IL-6 and IL-33 cytokines; and the tumor necrosis factor super family members, such as *Tnfrsf1a* and *Ntrk1* and their effector *Nfkb2*^24,38,42,43^ are well documented contributors to nociception and pain development (Fig. 9B). One of the immune DEGs—*CD74*—was revealed in every sensory neuronal preparation (Fig. 9A, Table 1). *CD74* is responsible for transport of MHC class II proteins^55^, which promote inflammatory and neuropathic pain^56^.

Other biological processes linked to female-selective sensory neuronal DEGs identified from different preparations are nervous system, vasculature and epithelium development (Figs. 4A,B, 8B). These DEG clusters contain many genes encoding growth factors and their receptors (*Mxra8*, *Arxes2*, *Notch3*, etc.), cell–cell and cell-extracellular matrix adhesion (*Islr*, *Bgn*, etc.), and channels and their modulators (*Pkib*, etc.; Figs. 4, 5, 9A). Growth factors and their receptors are modulators of nociceptive pathways and pain development. *Pdgf* regulates inflammatory pain^57^. *Fgf* and *Tgfb* signaling play a role in pain development during osteoarthritis, but they may be protective rather than pain promoting^58^. The *Egfr* signaling pathway has been implicated in nociception and pain based on human genetics as well as preclinical models where EGFR ligands promote pain via the EGFR and the mTOR pathway^59^. *Notch* signaling is involved in the development of pain via communication between sensory neurons and non-neuronal cells^60^. Channels and their modulators are likewise involved in regulation of nociceptive pathways. The *Hcn* family which plays a key role in underlying spontaneous activity and mechanical hypersensitivity after nerve injury^61^. Antagonism of *Hcn* with ZD7288 dose-dependently reduces mechanical hypersensitivity in a model of cervical radiculopathy pain^61^, and this antagonist is also effective for inflammatory pain^62^. *Kcnq* channels encode K^+^ channels that represent the M-current that regulates nociceptor signaling, especially at peripheral terminals^46,63^, and also plays an important role in generation of spontaneous activity after nerve injury^64^. The M current is also critical in mediating hyperalgesic actions of many G_q_-protein receptor activators such as bradykinin^65^ and protease activated receptors^66^. *Orai1* as a key component of store-operated calcium channels that regulate nociception via modulation of neuronal excitability and A-type potassium channels^67^. Delayed rectified potassium voltage-gated channels, *Kcnc2* and *Kcng2*, play key roles in action potential shaping and consequently in nociception transmission and pain plasticity^45^. *Kcnk1*8, a TWIK-related two-pore potassium channel is involved in regulating the resting membrane potential^68,69^ and has been linked to migraine with aura pathogenesis^70,71^. Finally, we identified higher expression of *Trpv4* in female TG sensory neuronal fractions and this ion channel plays an important role in pain sensitization that has been thoroughly studied^47,72^. Collectively these female-selective DEGs in sensory neurons obtained from a variety of preparations suggest a potential role of enhanced interactions between inflammatory cells and sensory neurons via specific signaling pathways that are involved in the development of chronic pain (Fig. 9B). An intriguing hypothesis that emerges from these findings is the suggestion of DEGs in female sensory neurons that could shift the balance between protective neuro-immunity and neuro-immunopathology that would be predicted to promote the transition of acute to chronic pain^73–75^.

As for genes with female-predominant expression, sets of male-selective DEGs depend on sensory neuron purification approaches and selection of ganglia. The highest numbers (369–370) of such DEGs (RPKM > 1; FC > 2; Pval < 0.05) were identified in Percoll purified DRG and TG neurons, and the lowest (24) were in DRG neurons innervating skin and thigh (Figs. 3D, 8C). Again, like for female-selective DEGs, there was substantial overlap in biological processes linked to sets of male DEGs (Figs. 6A,B, 8D). Two main biological processes related to male-selective DEGs are reactive oxygen species and protein metabolism, including proteasomal machinery and mitochondrial protein production (Figs. 6A,B, 8D). Protein metabolism is involved in recovery from nerve and tissue injuries^76^. An example of the important role of this pathway in neuropathy and neuropathic pain is inhibition of the proteasome with the chemotherapy agent bortezomib which causes distal axonopathy^49^, nerve degeneration^49^ and painful neuropathy^77,78^. In contrast, activation of the proteasome can lead to effective removal of misfolded proteins and changes in transcriptomic profiles^79^, and contributes to recovery from tissue injury^48^, nerve damage^49^ and inflammation^50^. The important roles of mitochondrial proteins and reactive oxygen species (ROS) metabolism, especially oxidative phosphorylation, in nerve and tissue degeneration/regeneration is highlighted by a robust literature of hundreds of papers^80,81^. Enhancing the mitochondrial oxidative phosphorylation process can be an effective approach for recovery from nerve damage and degeneration^82,83^ as well as painful neuropathies^84^. Excessive ROS is a common feature for many pain conditions including a variety of neuropathy conditions^76^. Additionally, some male-selective DEGs from Percoll purified sensory neurons could be linked to translational machinery processes (Figs. 6A,B), while DEGs from neurons innervating skin and muscle can cover cell–cell communication and peptide secretion processes (Fig. 8D). The translation machinery is one of key players in regulation of nociception pathways and development of pain conditions^85,86^.

Overall, our approaches identify a number of candidate biological processes that are associated with female-selective sensory neuronal DEGs, in particular in the TG, (Fig. 9B) and male-selective DEGs (Fig. 9C). These genes include very prominent players in nociception that could be involved in differential occurrences of pain disorders in men and women (Fig. 9B). In contrast, DEGs predominantly expressed in male sensory neurons may protect male sensory neurons from some of the consequences of nerve injury that are known to promote neuropathic pain (Fig. 9C). Based on our findings, we favor the hypothesis that sex differences in nociception may be partially based on DEGs in female and male sensory neurons. Our results advance our understanding of sex dimorphisms in nociceptive pathways and provide an informatic basis for further studies on regulation of sex-dependent gene expression plasticity in sensory neurons regulated by injury and/or by gonadal hormones^44,87,88^.

## Materials and methods

### Mouse lines and tissues

All animal experiments conformed to APS's Guiding Principles in the Care and Use of Vertebrate Animals in Research and Training, and to protocols approved by the University Texas Health Science Center at San Antonio (UTHSCSA) Animal Care and Use Committee (IACUC). We followed guidelines issued by the National Institutes of Health (NIH) and the Society for Neuroscience (SfN) to minimize the number of animals used and their suffering.

Eight-to-twelve-week-old naïve C57BL/6J (The Jackson Laboratory, Bar Harbor, ME) female in estrous phase and male mice were used for all described experiments. Estrous phase was determine by vaginal gavage as described previously^89^. In some fluorescent activated cell sorting (FACS) experiments, we used CGRP^cre/± ER^/Rosa26^LSL-tDTomato/+^ reporter mice^31^.

Left and right whole L3-L5 DRG and TG tissue biopsies were dissected after perfusion of mice with phosphate buffer pH 7.3 (PB). DRG and TG tissues were used either for total RNA isolation or for single-cell suspension generation. After perfusion of mice with PB, a 3 mm biopsy-puncher was set to take epidermal-dermal layers and with partial collection of fat cells located underneath of dermal layer of hindpaws. Hindpaw biopsies do not touch muscles. Paw tissues were used for total RNA.

### Sensory neuronal fraction isolation with Percoll gradient

To generate single-cell suspensions, whole L3-L5 DRG or TG were treated with 125 μg/ml liberase (Millipore-Sigma, St. Louis, MO) and 200 μg/ml dispase II (Millipore-Sigma, St. Louis, MO) in Hank’s solution for 60 min. Tissues was washed twice with DMEM/L-glutamate/5% fetal bovine serum (FBS) media and dispersed to single-cell conditions by pipette. Single-cell media solutions were filtered through 70 μm strainer and loaded on a 60%/30% Percoll (Millipore-Sigma, St. Louis, MO) gradient prepared in the same media. Gradient preparations were spun for 15 min at 1,000×*g* at 4 °C. The non-neuronal fraction that was accumulated between 0 and 30% Percoll was discarded, and neuronal fraction at the border between 30 and 60% Percoll was collected. The neuronal fraction was diluted eightfold and washed with media. Sensory neurons were collected by centrifugation for 5 min at 1,200×*g* at 4 °C.

### Isolation of DRG sensory neurons innervating hind paws and thigh muscles

Left and right male or female naïve mouse hind paws and thigh muscles were injected with 10 μl (each injection) of 1% WGA-Alexa Fluor™ 488 conjugate (WGA-488) dissolved in 1% DMSO. Two days post-injection, L3–L5 DRG were isolated and single-cell suspension was generated with enzymatic treatment as described above.

Flow cytometry was used to isolate labeled back-traced from skin and muscle DRG neurons. LIVE/DEAD viability staining was done with 7-AAD Viability Staining Solution diluted at 1:50. Labeled cells were sorted using 5 laser FACS Aria-IIIu cell sorter equipped with 130 μm nozzle.

Cell sorting was also used to assess the percentage and viability of CGRP-cre^+^ cells in DRG of CGRP^cre-ER/+^/Rosa26^LSL-tDTomato/+^ reporter male mice.

### RNA isolation and cDNA synthesis

RNA was isolated from male or female mouse whole DRG, whole TG and paw tissues, as well as single-cell sensory neuronal fractions. Fresh tissues were homogenized in Rn-easy solution using a Bead Mill Homogenizer (Omni International, Kennesaw, GA), while RNA isolation from single-cell sensory neuron suspension did not require homogenization. Sensory neuronal and tissue total RNA was extracted using Qiagen Rn-easy (Universal Mini Kit) as was previously described^90^. If the preparation generated > 10–20 ng RNA, then RNA quality and integrity were checked using Fragment Analyzer Agilent 2,100 Bioanalyer RNA 6,000 Nano chip (Agilent Technologies, Santa Clara, CA). For small amounts of RNA (< 10 ng), quality of RNA was accessed after cDNA preparation.

#### Preparation of cDNA library and RNA-seq from tissue RNA

Approximately 500 ng total RNA was used for cDNA library preparation with oligo-dT primers by following the Illumina TruSeq stranded mRNA sample preparation guide (Illumina, San Diego, CA).

#### Preparation of cDNA library and RNA-seq from Percoll enriched sensory neuronal fraction

The first step in the workflow involved the depletion of rRNA by hybridization of complementary DNA oligonucleotides, followed by treatment with RNase H and DNase to remove rRNA duplexed to DNA and original DNA oligonucleotides, respectively. Following rRNA removal, the mRNA was fragmented into small pieces using divalent cautions under elevated temperature and magnesium. The cleaved RNA fragments were copied into first strand cDNA using reverse transcriptase and random primers. This was followed by second strand cDNA synthesis using DNA Polymerase I and RNase H. Strand specificity was achieved by replacing dTTP with dUTP in the Second Strand Marking Mix (SMM). The incorporation of dUTP in second strand synthesis effectively quenches the second strand during amplification, since the polymerase used in the assay will not incorporate past this nucleotide. These cDNA fragments then went through an end repair process, the addition of a single ‘A’ base, and then ligation of the adapters. The products were then purified and enriched with PCR to create the final RNA-seq library.

#### Preparation of cDNA library and RNA-seq from Percoll enriched sensory neuronal fraction

Sensory neurons isolated by sorting DRG neurons back-traced and labeled with WGA-488 generated < 10 ng RNA. Hence, RNA-seq cDNA libraries were prepared using oligo dT according to SMART-seq-2 protocol^91,92^, with the following modifications: 1: All primers were designed with 5′ biotin modifications to prevent primer concatenation; 2: PCR preamplification to 10–12 cycles; 3: Two rounds beads cleanup with 1:1 ratio after cDNA synthesis, and 4: 0.6–0.8 dual beads cleanup for Nextera XT DNA-seq library purification.

All RNA-seq cDNA libraries were subjected to quantification, pooled for cBot amplification and subsequent 50 bp single read sequencing run with Illumina HiSeq 3,000 platform (Illumina, San Diego, CA). Each group have n = 3–5 samples. Depth of reads was 30–50 × 10^6^ bp for each sample.

### RNA seq transcriptomic data generation, analyses and statistics

Sequencing data from all samples were processed in the same way. After the sequencing run, de-multiplexing with CASAVA was employed to generate the FastQ file for each sample. The combined raw reads were aligned to mouse genome build mm9/UCSC hg19 using TopHat2 default settings^93,94^. The BAM files obtained after alignment were processed using HTSeq-count to obtain the counts per gene, and then converted to RPKM (Read Per Kilobase of gene length per Million reads of the library)^95^. Then DESeq was used to identify differentially expressing genes (DEGs) after performing median normalization^96^. Quality control statistical analysis of outliers, intergroup variability, distribution levels, PCA and hierarchical clustering analysis was performed to statistically validate the experimental data. Multiple test controlling was performed with Benjamini–Hochberg procedure and adjusted p-value (Padj) was generated.

Three criteria have been used for selecting DEGs for the further analysis: expression levels (i.e. RPKM), fold-change (FC) and statistically significant DEGs (i.e. Pval or Padj). Selection criteria vary depending on cDNA library preparation. These selection criteria and their justifications and rationales are in the text. Venn diagrams were generated using https://bioinfogp.cnb.csic.es/tools/venny/. Genes were clustered according to biological processes using the PANTHER software (https://www.pantherdb.org/). Pathway analysis was performed with Ingenuity Pathway Analysis (IPA, Qiagen, CA).

### Quantitative real-time PCR

Quantitative real-time PCR (RT-PCR) was performed on independently isolated sensory neuronal fraction from mouse male and female TG and DRG using Percoll purification protocol (see above). Quantitative RT-PCR procedure has essentially been performed as described^90^. Briefly, cDNA from RNA was prepared using the Superscript III First Strand Synthesis kit (Invitrogen). Amplification of target sequences was detected on ABI 7,500 Fast RTPCR system (Applied Biosystems, Foster City, CA) using TaqMan Fast Universal PCR Master Mix. The reactions were run in triplicates of 25 μl. The endogenous control for each individual gene expression assay was mouse GAPDH with Mm03302249_g1 primers (ThermoFisher Sci.), which express equally in female and male DRG and TG neurons. Specific primers were Mm00658576_m1 for Cd74, Mm01700423_m1 for Islr, Mm01191753_m1 for Bgn, Mm00470429_g1 for Mxra8, Mm00435846_m1 for Pkib and Mm00547130_s1 for Arxes2. For quantitative analysis, comparative delta-delta Ct was used to normalize the data based on the endogenous reference, and to express it as the relative fold change, after the exclusion criteria were verified by comparing primer efficiencies^90^.

### Ethical approval and informed consent

All experimental protocols were approved by the UTHSCSA IACUC committee. Protocol numbers are 20150100AR and 20180001AR.

## Supplementary information

Supplementary Excel File 1.

Supplementary Excel File 2.

Supplementary Excel File 3.

Supplementary Excel File 4.

Supplementary Excel File 5.

## Data Availability

RNA-seq data has been deposited to GSE121645, GSE135909 and GSE153269. Supplementary excel files for the raw gene counts per gene in all of our sequencing experiments. These supplementary files are *Whole DRG (Female Vs Male)* for sequencing from whole DRG; *Hindpaw skin (Female vs Male)* for sequencing from epidermis and dermis layer of hindpaw skin; *DRG neurons by Percoll (Female vs Male)* for sequencing from Percoll purified DRG neurons; *TG neurons by Percoll (Female vs Male)* for sequencing from Percoll purified TG neurons; and *WGA-488+ (skin-thigh innerv) DRG neurons (Female vs Male)* for sequencing from a mix of DRG neurons innervating hindpaw skin and thigh muscle.
